# Comparison of the brain development trajectory between Chinese and U.S. children and adolescents

**DOI:** 10.3389/fnsys.2014.00249

**Published:** 2015-02-02

**Authors:** Wanze Xie, John E. Richards, Du Lei, Kang Lee, Qiyong Gong

**Affiliations:** ^1^Department of Psychology, Institute for Mind and Brain, University of South CarolinaColumbia, SC, USA; ^2^Huaxi MR Research Center (HMRRC), Department of Radiology, West China Hospital of Sichuan UniversityChengdu, China; ^3^Department of Human Development and Applied Psychology, Dr. Eric Jackman Institute of Child Study, University of TorontoToronto, ON, Canada

**Keywords:** Chinese children and adolescents, brain development, structural MRI

## Abstract

This current study investigated brain development of Chinese and American children and adolescents from 8 to 16 years of age using structural magnetic resonance imaging (MRI) techniques. Analyses comparing Chinese and U.S. children brain/head MR images were performed to explore similarities and differences in the trajectory of brain development between these two groups. Our results revealed regional and age differences in both brain/head morphological and tissue level development between Chinese and U.S. children. Chinese children's brains and heads were shorter, wider, and taller than those of U.S. children. There were significant differences in the gray matter (GM) and white matter (WM) intensity between the two nationalities. Development trajectories for cerebral volume, GM, and several key brain structures were also distinct between these two populations.

## Introduction

Structural magnetic resonance imaging (MRI) provides a non-invasive method to investigate the anatomy and physiology of human brain development. Over the past two decades, a great number of MRI scans from healthy children and adolescents have been acquired. Reports from these works have contributed to our understanding of the trajectory of brain development throughout childhood and early adulthood (Jernigan et al., 1991; Caviness et al., 1996; Giedd et al., 1996a,b, 1999; Reiss et al., 1996; Sowell and Jernigan, 1998; Courchesne et al., 2000; Sowell et al., 2004; Lenroot et al., 2007). These studies have revealed regional brain maturational changes continuing throughout childhood and adolescence. It should be noted, however, that the existing data were collected from children drawn from Western European or North American populations. It is entirely unclear as to whether the current knowledge about brain structural development reflects a universal pattern of development or a pattern specific to one cultural-racial group. The latter possibility cannot be ruled out due to the fact that MRI studies with adults have found brain morphometric and volumetric differences between Asian and North American adults (Lee et al., 2005; Tang et al., 2010). To our knowledge, very few neuroimaging studies have examined brain development for Asian populations (Guo et al., 2007, 2008), and no study has directly compared the brain development patterns and brain anatomical features between Asian and American child populations. To bridge this important gap in the literature, we investigated the brain development of Chinese children and adolescents from 8 to 16 years, and explored differences in developmental trajectories and anatomical features between Chinese and U.S. children and adolescents.

There is a large body of literature examining brain development from early childhood to adolescence in North American populations. Total cerebral volume follows an inverted U-shape developmental trajectory peaking at early adolescence. Lenroot et al. (2007) study is the largest pediatric neuroimaging study to date with 829 scans from 387 subjects, age 3–27 years. They found that total cerebral volume develops from early childhood to adolescence and peaks at age 10.5 in females and 14.5 in males. Postmortem studies also show that total brain weight increases dramatically during the first 5 or 10 years of life, but increases slowly in the late teens and early 20 s (Dekaban and Sadowsky, 1978; Ho et al., 1980). The overall developmental trajectory of gray matter (GM) also follows an inverted U-shape with its volume peaking at different times for different lobes (Pfefferbaum et al., 1994; Reiss et al., 1996; Giedd et al., 1999; Lenroot et al., 2007; Sanchez et al., 2012a). For instance, Jernigan and Tallal (1990) reported that children aged 8 and 10 years had significantly more cortical GM relative to cerebral size than did young adults. Giedd et al. (1999) and Lenroot et al. (2007) found that GM development peaked at around 8- to 9-years old. The changes of GM also show regional heterogeneity. For example, Giedd et al. (1999) found that cortical GM in the frontal and parietal cortices increases during pre-adolescence to a maximum amount roughly at puberty (age 12 years for males, 10–11 years for females). However, temporal cortex GM increases until about 16 years for males and females, and occipital GM increases linearly through childhood and adolescence, without evidence of significant decline (Giedd et al., 1999). By contrast, there is a linear increase in overall cerebral white matter (WM) throughout childhood and young adulthood (Caviness et al., 1996; Rajapakse et al., 1996; Lenroot et al., 2007; Sanchez et al., 2012a). A consistent finding across these studies is that WM increases throughout early childhood and young adulthood with males having a steeper rate of increase during adolescence. Unlike the lobar differences seen in GM, the change in WM is similar across cortical lobes. For example, frontal and parietal lobes show highly significant increases in WM volumes throughout this time period (Giedd et al., 1999; Sowell et al., 2002). The more ventral regions in the temporal lobes appear to change less dramatically throughout childhood and adolescents (Jernigan et al., 1991; Cowell et al., 1992; Giedd et al., 1999; Sowell et al., 2002).

Early morphometric evidence for head shape differences between Asian and Western European or North American populations has been reported in adult anthropometric studies (Beals et al., 1984; Farkas, 1994; Ball et al., 2010). Traditional anthropometrical measurements of human head and body used measuring tapes and calipers while more recent studies applied new digital methods to collect 3D landmark coordinates that can be used for statistical shape analysis. Ball et al. (2010) combined and analyzed two recent 3D anthropometric surveys collected in North America, Europe, and China, separately. The researchers quantified and compared the head shape and size between Chinese and Caucasians. Results demonstrated that Chinese heads are generally rounder than Caucasian counterparts, with a flatter back and forehead. With respect to head size, Chinese heads are generally shorter, wider, and smaller in height than Caucasian heads.

Differences in brain and head shapes and sizes between Asian and Caucasian groups have also been documented in neuroimaging studies. Kochunov et al. (2003) used MRI to detect differences in brain shape between Caucasians and Asians. Anatomical differences between these two groups were found in the gyri in the frontal, temporal and parietal lobes. Lee et al. (2005) created a MRI brain template based on Korean adults. They found that a standard Korean brain template was 10% shorter in length, 9% lower in height, and 1% greater in width compared to the International Consortium for Brain Mapping (ICBM-152) template using 152 normal North American adult participants (Mazziotta et al., 2001). Similar findings were reported by Tang et al. (2010), who created the Chinese_56, an adult MRI brain template based on 56 male Chinese adult MR images. Tang et al. (2010) found that the Chinese_56 was shorter (168.77 vs. 177.00 mm), wider (144.39 vs. 136.00 mm), and notably lower in height (110.64 vs. 124.00 mm) than the ICBM-152. In addition to these comparisons between the Chinese_56 and the ICBM-152 templates, Tang et al. (2010) performed direct comparisons of brain morphological features (length, width, height) between MRIs of 35 Chinese and 35 North American adult male brains. The 35 Chinese male subjects were randomly chosen from the database (63 Chinese male adults) they used to construct the Chinese_56 template, and the 35 North American male subjects were randomly selected from the ICBM database (http://ida.loni.ucla.edu). The results from direct comparisons were inconsistent with their measurements of brain templates in terms of the brain height. Direct comparison showed that Chinese adult brain was taller than North American brain while the measurements of height between the Chinese56 and ICBM-152 templates had the inverse result. Comparison of brain regional volumes between Chinese and North American adults was also performed in Tang et al. (2010). For all 35 Chinese and 35 North American brain MRI scans examined, 56 brain structures were automatically obtained including 50 cortical brain structures, 4 sub-cortical brain structures, the brain stem, and the cerebellum. Overall, their study showed that the Chinese and North American brains were significantly different in volume in many structures (e.g., the left middle orbitofrontal gyrus, left gyrus rectus, and right insular cortex, see Tang et al., 2010 for more details). No specific comparisons for GM and WM intensity were conducted.

Very few studies have examined the brain development of Chinese children and adolescents, and little is known about how it might differ from North American age-related populations. Guo et al. (2007, 2008) conducted the only systematic studies of Chinese children's brain development. MRIs for 158 healthy 7- to 23-year-old Chinese children, adolescents, and young adults were collected. No North American participants were included in their studies. Their results revealed that overall GM volume decreased linearly with age while overall WM volume increased linearly with age. Effects of age on the regional variations were also found in their studies. For example, positive correlations between GM volume and age were observed in subcortical (e.g., hippocampus, amygdala) and some cortical regions (e.g., inferior temporal gyrus, left fusiform gyrus) while negative correlations were found in many other regions (e.g., bilateral parietal lobe). In terms of WM, age-related linear increases were found in some brain regions (e.g., internal capsule, inferior longitudinal fasciculus). Some of the findings were consistent with previous studies on U.S. children and adolescents (e.g., overall increases of white matter volume, reductions in GM volume in parietal lobe) while some were different with previous volumetric studies (e.g., overall decreases of GM volume).

Whereas Guo and colleagues' work provides information about the developmental trajectory of a non-Western group, several limitations still exist in the literature. The current study addressed several issues by making the following improvements. First, direct comparisons between age-related Chinese and U.S. children and adolescents were conducted to ascertain whether the similarities and differences reported in the existing studies are genuine cross-ethnic differences. Second, the sample size was expanded, which was necessary as Guo and her colleague only scanned 158 subjects, of which one third were young adults. Third, in addition to brain volume assessment, morphometric analysis were added to test brain/head shape and size changes. Fourth, GM development was not only studied for the global volume but also for different major brain lobes (e.g., frontal lobe, temporal lobe).

We specifically examined the brain development patterns of Chinese children and adolescents ranging from 8 to 16 years old, and directly compared their brain development with age-related U.S. cohorts. We collected MR images for both Chinese and U.S. children and adolescents. Based on previous research, differences in brain development were expected between Chinese and U.S children and adolescents. Specifically, we hypothesized that (1) Chinese children's brain and head development would differ in morphological features, i.e., in shape and size. Chinese children's brains and heads may be shorter, wider, and taller than those of U.S. children. (2) Given morphological differences between Chinese and U.S. children, we expected volumetric differences (e.g., total brain and head volume, overall GM and WM intensity development) in brain development between these two populations. (3) We expected that the development of Chinese and U.S. children's brain volumes and GM intensity may be distinct in regional brain structures, especially in those regions that were highlighted in the Tang et al. (2010) adult study.

## Methods

### Participants

The MRIs for Chinese participants were collected from 133 children and adolescents ranging from 8 through 16 years of age (50 F/83 M). These participants were recruited from a local community in Sichuan province, China. The gender and number of subjects within each age group are listed in Table 1.

**Table 1:** **Demographic Information for Chinese and US participants**.

**Age group**	**Nationality**	**Total *N***	**Gender (#Male)**
8 Years	CN	16	12
	US	19	11
9 Years	CN	14	12
	US	8	4
10 Years	CN	8	8
	US	16	9
11 Years	CN	13	10
	US	12	6
12 Years	CN	23	13
	US	15	11
13 Years	CN	18	12
	US	29	15
14 Years	CN	21	7
	US	30	17
15 Years	CN	8	3
	US	8	4
16 Years	CN	12	6
	US	12	6
**GROUPED IN TWO YEARS INCREMENT**			
8 Years	CN	16	12
	US	19	11
9–10 Years	CN	22	20
	US	24	13
11–12 Years	CN	36	23
	US	27	17
13–14 Years	CN	39	19
	US	59	32
15–16 Years	CN	20	9
	US	20	10
Total	CN	133	83
	US	149	83

There were 149 (66 F/83 M) healthy, age-related participants of U.S. nationality. The MR images for the U.S. children and adolescents were collected from (1) participants at the University of South Carolina McCausland Center for Brain Imaging (USC-MCBI) (Sanchez et al., 2012b), and (2) normal controls from the Autism Brain Imaging Data Exchange (ABIDE) (Di Martino et al., 2014).

The Institutional Review Boards (IRBs) of the West China Hospital in Sichuan University and the University of South Carolina approved this study. All participants' parent(s) signed a written consent form on behalf of the children and adolescents enrolled in the study.

### MRI data acquisition

The Chinese children's MRI scans were collected with two MRI scanners in the Huaxi MR Research Center of the West China Hospital of Sichuan University in Chengdu, Sichuan, China. The majority of the subjects (*N* = 108) were scanned using a 3.0T Siemens Trio Scanner. These high-resolution 3-dimensional T1-weighted images were acquired using a MPRAGE sequence with the following parameters: *TR*/*TE*/*TI* = 1900/2.26/900 ms, Flip angle = 9°, 176 axial slices with thickness = 1 mm, axial FOV = 25.6 × 25.6 cm^2^ and data matrix = 256 × 256. The remaining 22 subjects were scanned with a 3.0T GE SIGNA MRI scanner. High-resolution 3-dimensional T1-weighted images were acquired using a spoiled gradient recalled (SPGR) sequence with the following parameters: *TR*/*TE* = 8.5/3.4 ms, Flip angle = 12°, 156 axial slices with thickness = 1 mm, axial FoV = 24 × 24 cm^2^ and data matrix = 512 × 512.

The U.S. age-related MRIs from the MCBI and ABIDE databases were collected with 3.0T scanners. The MRI data from the USC-MCBI were collected on a Siemens Medical System 3T Trio with a 3D T1-weighted MPRAGE RF-spoiled rapid flash scan in the sagittal plane with the following parameters: *TR* = 2250 ms, *TE* = 4.52 ms, flip angle = 9°, FoV = 256 × 256 mm, matrix size = 1 × 1 × 1 mm^3^ (the sagittal dimension of the T1W ranged from 160 to 212 slices). The scans had sufficient FoV to cover from the top of the head down to the neck. For more information on MRI acquisition procedures at the USC-MCBI (see Sanchez et al., 2012b).

The ABIDE MRIs came from a variety of sites and scanners. All MRIs were done as MPRAGE scans, on a 3.0T strength scanner, with slice thickness of 1.0–1.3 mm, and sufficient FoV to cover the entire brain. The scanners and TR/TE used for the ABIDE MRIs include Siemens Magneton TrioTim (*TR* = 1230, *TE* = 1.7; *TR* = 1590, *TE* = 2.7, *TR* = 2300, *TE* = 2.8; *TR* = 2300, *TE* = 2.9; *TR* = 2300, *TE* = 3.6), Siemens Magneton Verio (*TR* = 1800, *TE* = 3.1), Siemens Magnetom Allegra (*TR* = 2100, *TE* = 3.9; *TR* = 2500, *TE* = 2.7; *TR* = 2530, *TE* = 3.3), GE MR750 (*TR* = 2000, *TE* = 4.3), GE Signa (*TR* = 9000, *TE* = 1.8), Phillips Achieva (*TR* = 8000, *TE* = 3.7; *TR* = 8500, *TE* = 3.9), Phillips Intera (*TR* = 9600, *TE* = 4.6). The USC-MCBI (3T) files were read from DICOMM files to compressed NIFTI format (http://nifti.nimh.nih.gov/). The ABIDE files came from the LONI site (loni.UCLA.edu) in compressed NIFTI format.

### File preparation and tissue classification

The MR images were prepared for processing in two steps. First, the brains were extracted from the whole-head MRI volume using the FSL computer programs (Jenkinson et al., 2012). An automated bash script using the FSL tools (Smith et al., 2004; Woolrich et al., 2009) completed this task. Second, the FSL “betsurf” tool (Jenkinson et al., 2005) was used to create an inner skull mask in order to calculate the inner skull volume. Third, the individual participant MRI volumes were classified into GM, WM, and CSF. The FSL FAST procedure, “FMRIB's Automated Segmentation Tool” (Zhang et al., 2001) was used for T1W scan segmentation. This was completed with the FSL FAST program without prior classification volumes and resulted in a set of partial volume estimates (PVE) for GM, WM, and CSF, for each participant's MRI volume.

### Participant macroanatomical atlases

Two atlases were constructed on the individual participant's MRIs. One atlas was constructed using the LONI Probabilistic Brain Atlas segmentation (40 individuals, 56 manually delineated areas including 50 cortical structures; LPBA40) (Shattuck et al., 2008). This was done with an established procedure that used the 40 adult-manually-segmented heads from the LPBA40 atlas and registered these heads to an individual participant, transforming the adult volume to the individual's MRI space (for details on the use of this method with 2-year-olds see Gousias et al., 2008; for infants see Phillips et al., 2013). The 40 atlases were then fused in a majority vote procedure (see more detail on this procedure in Gousias et al., 2008) to identify a macro-anatomical area for each brain voxel in the individual MRI volume. The resulting atlas identifies the majority-voted brain segment for each voxel of the individual brain. The second atlas was a lobar atlas that identified the major cerebral lobes (e.g., frontal lobe, temporal lobe). The lobar atlas was constructed by manual segmentation of the major lobes on an average MRI template created with young US adults (Sanchez et al., 2012b). The individual participant MRI was linearly registered to the average MRI template, and the lobar atlas was transformed by the linear registration matrix into the individual participant's MRI space. The procedures for generating macro-anatomical and lobar atlases have been recently used across a range of individual infant MRIs and age-appropriate templates from 3 to 12 months of age (Phillips et al., 2013).

### Locations on MRIs and brain and head morphometric measurement

External scalp and internal anatomical locations were manually marked on individual MRIs. The MRIcron program (Rorden, http://www.mccauslandcenter.sc.edu/mricro/mricron/) was used to display the MRI and create individual masks on these locations. Two locations were identified inside the head, including the anterior commissure and posterior commissure. Several locations were identified on the scalp, including the nasion, inion, and left and right pre-auricular skull locations.

Brain and head morphological features were calculated from these markers. The distances between nasion and inion, left and right pre-auricular locations, top of the head to PC, and AC and PC were assessed as head length, width, height, and AC-PC distance. The distances from the front of the brain to the back of the brain on the AC-PC line was measured as the brain length. The brain width was measured as the distance between the left edge and the right edge of the brain on the line normal to the AC-PC. The distance between the top of brain and the PC was measured as the brain height on a line normal to the AC-PC line.

### Measurements of brain and head volumes

Brain, skull, and head volume were calculated. The brain volume was calculated based on the number of non-zero voxels in the extracted brain. Inner skull volume was calculated as the number of non-zero voxels in the “inner skull mask” created with the betsurf tool (Jenkinson et al., 2005). The “scalp mask” created with the betsurf tool was used to calculate head volume, with the following procedure. One semi-circumference reference plane was drawn from the left preauricular point through the nasion to the right preauricular point, and a second was drawn from the left preauricular point through the inion to the right preauricular point (MATLAB) and the MRI voxels in the scalp mask above these planes were defined as the head volume.

Volumes of the global and local brain features were calculated from the segmented GM/WM PVE files and the participant's atlas. The global GM or WM volume was calculated from the sum of the PVE values multiplied by the number of voxels in the brain. For the segmented lobar measures, we first masked the GM or WM PVE files with the segmented atlas section, and then calculated the volume of each brain lobe. Finally, a similar procedure was completed for the LPBA40 atlas.

### Analysis strategy

To test our first hypothesis, we examined the morphological differences in head and brain MR images between Chinese and U.S. children and adolescents with MANOVA and univariate analyses. These analyses were performed using the SAS program (SAS Institute Inc., Cary, NC, USA). The MANOVA was examined the effects of age and nationality on children's global brain and head shape as a composite of several morphological features. Four dependent variables that represent brain morphological features were tested as a group in the MANOVA. Univariate analyses (i.e., ANOVAs) were conducted to examine differences in specific brain features. Age and nationality were examined as independent variables in order to determine how the brain morphology changes as a function of age and nationality groups. Age was tested as a categorical variable to analyze differences between specific age groups (e.g., 8 vs. 10).

Our second hypothesis tested volumetric differences (e.g., total brain and head volume, overall GM and WM intensity) in brain development between these two populations. General linear model (GLM) analyses were performed to evaluate the effects of age, nationality, and gender on global cerebral volumes, inner skull volume, GM volumes, and WM volumes. Although we had uneven numbers of male and female participants in the younger age groups, gender was analyzed as a factor so that our results would be comparable to previous research that examined these variables and showed gender as factor influencing brain development (e.g., Giedd et al., 1999; Lenroot et al., 2007). Age was analyzed as a categorical variable, and participants were grouped in one-year increments.

Our third hypothesis tested regional GM and volume development and differences between Chinese children and adolescents' brains and U.S. age-related participants' brains. Therefore, the effects of age and nationality on GM intensity and regional volume in major brain lobes (e.g., frontal lobe, temporal lobe) and 50 cortical brain structures (LPBA40) were examined using GLM analyses. Analyses were expected to be more vulnerable to the effects of outliers when looking at regional structural volumes than overall brain volumes. Participants were grouped in 2-year increments, which increased the number of subjects in each age group. The focus of the current study was on the effects of nationality and its interaction with age on brain development. We had limited numbers female Chinese subjects in the young age groups, so we added gender as a factor to control for its effects, but do not report the results of the gender effect from the analyses examining the third hypothesis.

## Results

### Hypothesis I: morphological brain/head changes between chinese and U.S. children

The development of brain morphology in Chinese and U.S. children was examined first using multivariate analyses. A MANOVA was conducted to analyze the composite of brain anatomical features (brain length, width, height, and ac-pc distance) as a function of age and nationality. The multivariate result was significant for both age, Wilks' *L* = 0.873, *F*_(16, 816)_ = 2.32, *p* < 0.01, and nationality, Wilks' *L* = 0.616, *F*_(4, 267)_ = 41.58, *p* < 0.001. The interaction between age and nationality was not significant, Wilks' *L* = 0.920, *F*_(16, 816)_ = 1.41, *p* = 0.129.

**Figure 1:**
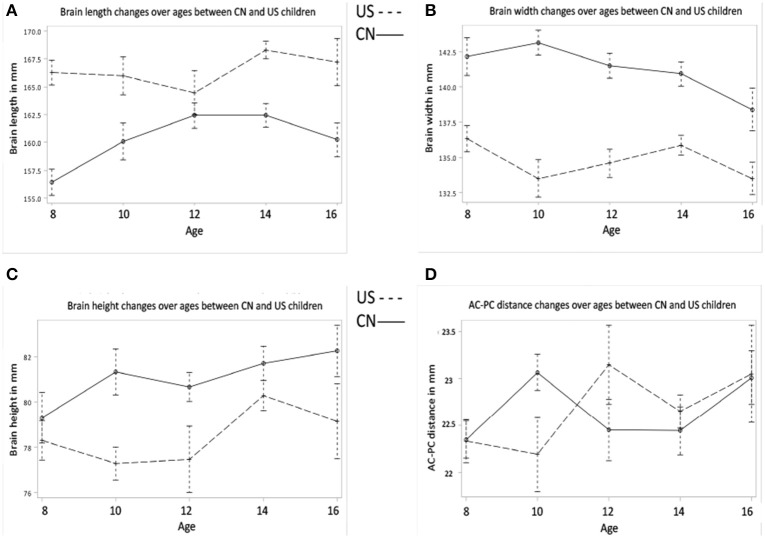
**Brain morphology develops as a function of age and nationality. (A)** Brain length changes over ages for Chinese and U.S. children. **(B)** Brain width changes over ages between Chinese and U.S. children. **(C)** Brain height changes over ages between Chinese and U.S. children. **(D)** AC-PC distance changes over ages for both Chinese and U.S. children. Note that brain length of Chinese children is smaller than that of U.S. children, but brain width and height of Chinese children are greater than those of their U.S. age-related peers.

Figures 1A–D shows the effects of age and nationality on the development of the four brain morphological features. The univariate ANOVAs for age and nationality are shown in Table 2. In summary, the Chinese children's brains were significantly shorter, wider, and taller than the U.S. children's brains (Figures 1A–C). There was no difference in the AC-PC distance (Figure 1D). In addition to the nationality effects, both Chinese and U.S. children showed increases over these ages in brain length, height, and AC-PC distance, but no significant change over age in brain width.

**Table 2:** **Hypothesis I: Morphological features development for Chinese and US children brain and head**.

**Measurement**	**Figures**	**Nationality**	**Age**
Brain length	Figure 1A	*F*_(1)_ = 66.31^***^	*F*_(4)_ = 2.89^*^
		US > CN	CN: inverted U
			US: U-shape
			But overall increase for CN, US
Brain width	Figure 1B	*F*_(1)_ = 71.18^***^	
		CN > US	
Brain height	Figure 1C	*F*_(1)_ = 10.95^**^	*F*_(4)_ = 3.86^**^
		CN > US	Overall increase for both CN and US with similar patterns
ac-pc distance	Figure 1D		*F*_(4)_ = 2.66^*^
			Overall increase for CN and US
Head length	Figure 2A	US > CN^***^	*p* < 0.001
			Overall increase for both CN and US
Head width	Figure 2B	CN > US^***^	*p* <.001
			Overall increase for both CN and US
Head height	Figure 2C	CN > US^***^	

Significant level:

* p < 0.05;

** p < 0.01;

*** p < 0.001;

empty cell refers to non-significant results.

**Figure 2:**
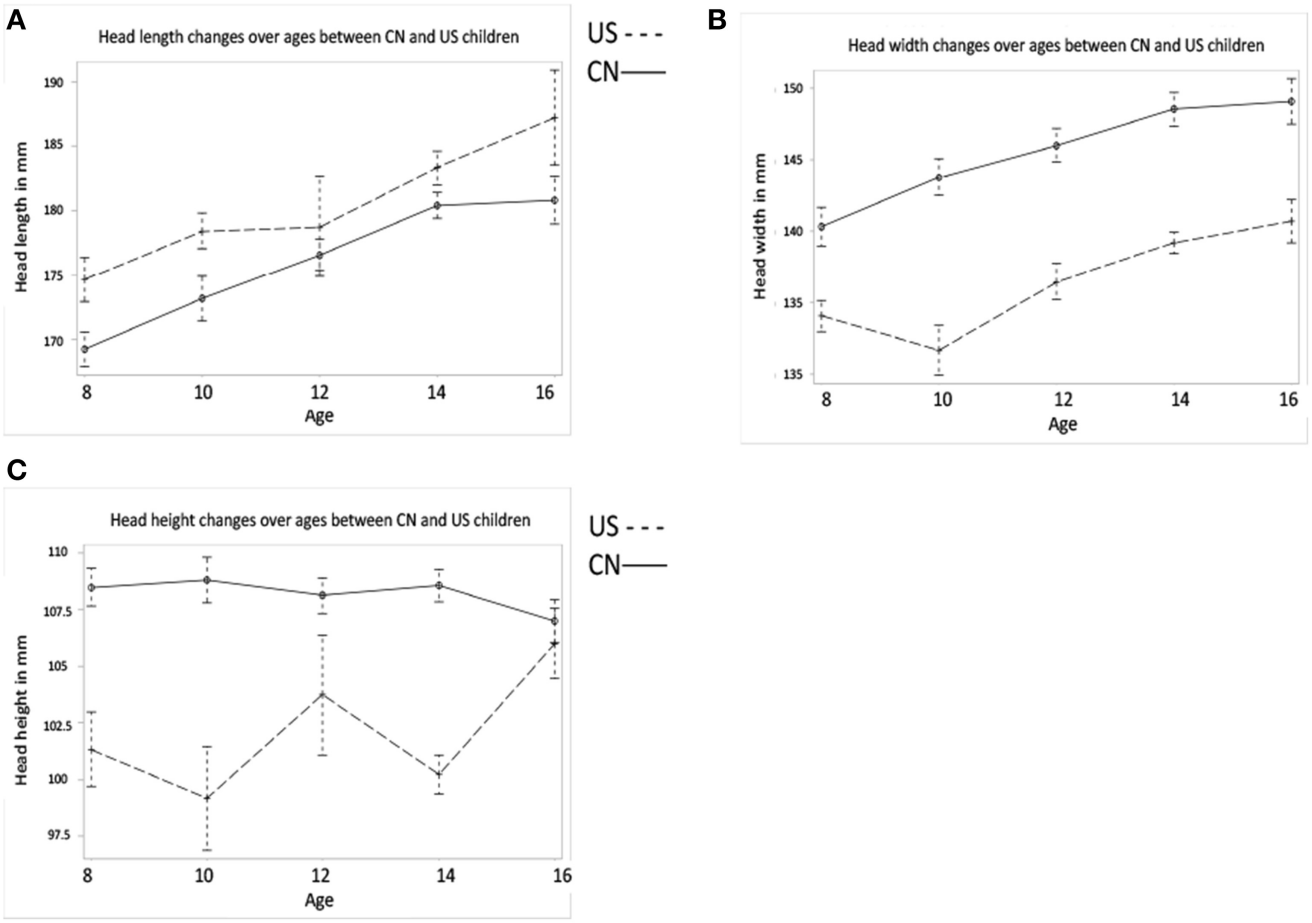
**Head morphology develops as a function of age and nationality. (A)** Head length development for the two nationalities. **(B)** Head width development for two nationalities. **(C)** Head height development for two nationalities. Morphological features of Chinese children' head also differ from their U.S. cohorts. In specific, Chinese children's head is shorter, wider, and higher than U.S. children's head.

Similar multivariate and univariate statistical analyses were conducted to examine head morphological development (head length, width, and height) as a function of age and nationality. MANOVA results revealed significant effects for both age [Wilks' *L* = 0.740, *F*_(12, 688)_ = 6.92, *p* < 0.001] and nationality [Wilks' *L* = 0.616, *F*_(3, 260)_ = 68.47, *p* < 0.001] on the composite of head features. No interaction was found, Wilks' *L* = 0.936, *F*_(12, 688)_ = 1.46, *p* = 0.134. All univariate results for head features are listed in Table 2. The changes in head morphological development were consistent with the changes in brain morphology. The Chinese children's head was shorter, wider, and taller than the U.S. children's head. One inconsistent finding was that both Chinese and U.S. head width increased with age (Figure 2B), but their brain width either declined with age (Chinese) or showed no clear increase or decline pattern (U.S.) (Figure 1B).

### Hypothesis II: head, intracranial, brain MRI volumes, and GM and WM changes

**Figure 3:**
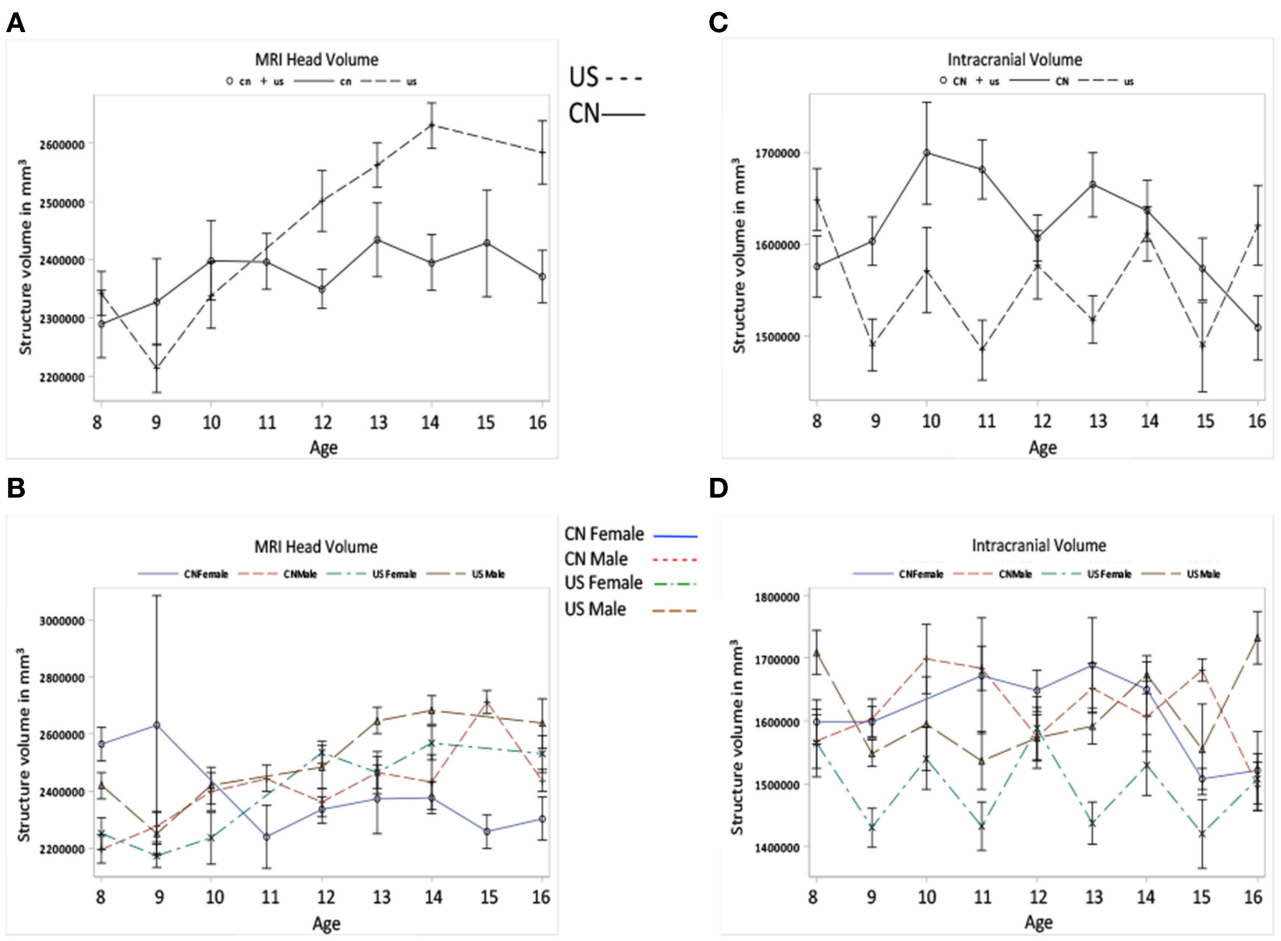
**Global head and intracranial volume development. (A)** MRI head volume development for Chinese and U.S. children. **(B)** The Intracranial volume development for the two nationalities. Note that **(A,B)** show the effects of age and nationality on MRI head and intracranial volume changes. **(C,D)** also show the MRI head and intracranial volume development but the factor of gender is included.

**Figure 4:**
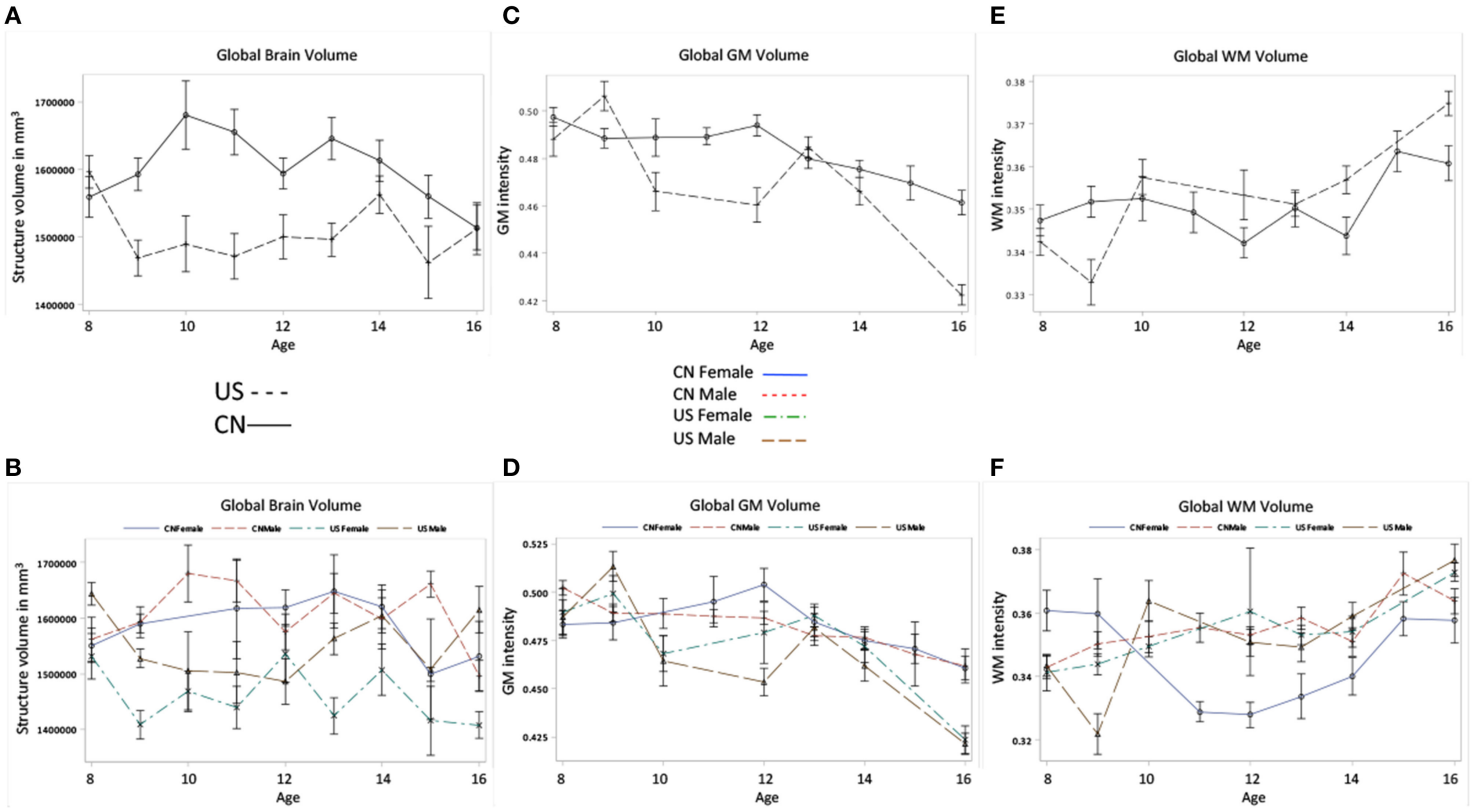
**Total cerebral, GM, and WM volumes change as the function of age, nationality and gender. (A)** Global brain volume development for Chinese and U.S. children. **(B)** The global brain volume development for Chinese and U.S. male and female children. **(C)** Global GM volume development for Chinese and U.S. children. **(D)** Global GM volume development for Chinese and U.S. male and female children. **(E)** Global WM volume development over ages for Chinese and U.S. children. **(F)** Global WM volume development over ages for Chinese and U.S. male and female children.

**Figure 5:**
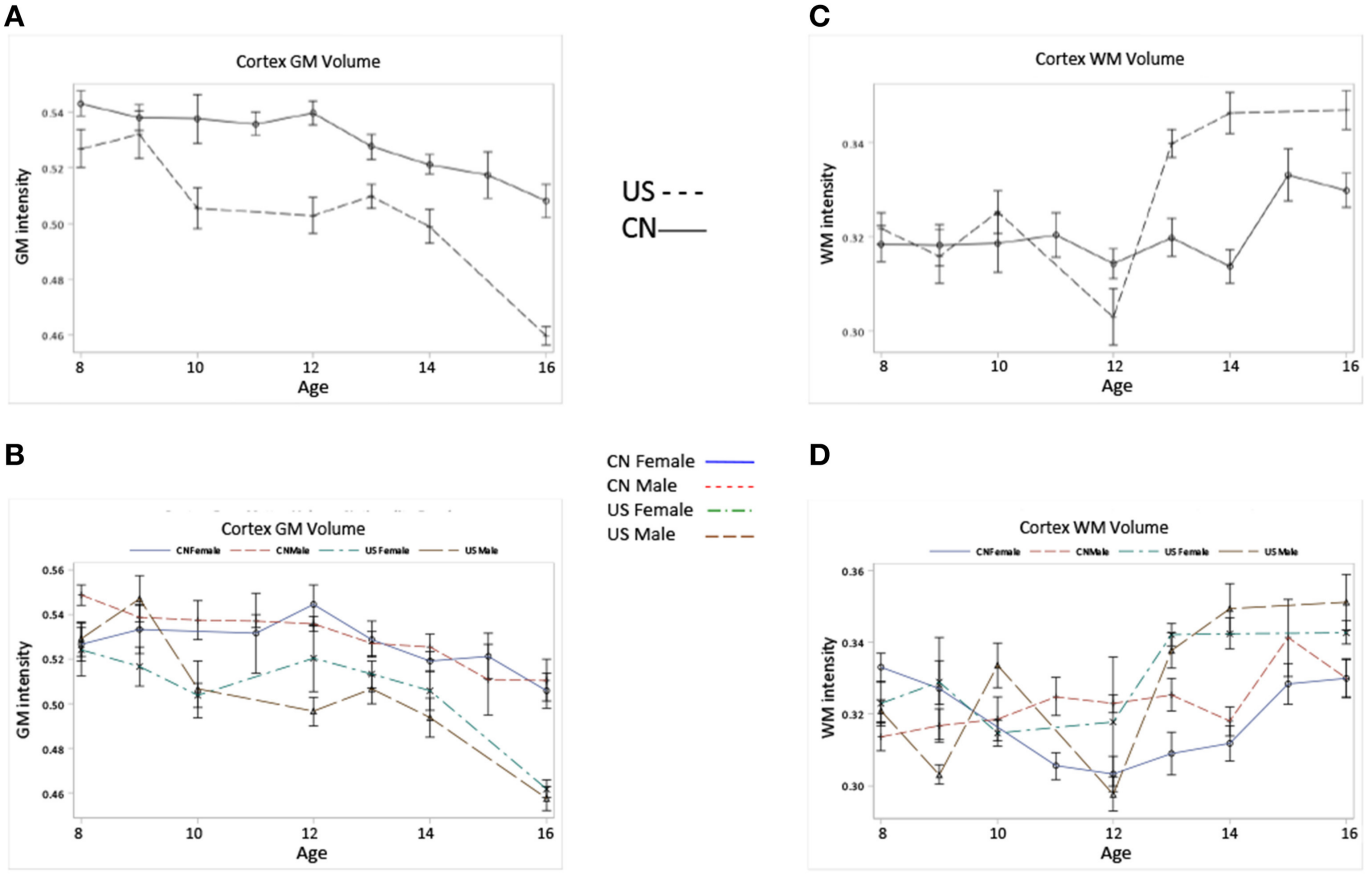
**Cortical GM, and WM volumes change as the function of age, nationality and gender**. **(A)** Cortical GM volume development for Chinese and U.S. children. **(B)** Cortical GM volume development for Chinese and U.S. male and female children. **(C)** Cortical WM volume development for Chinese and U.S. children. **(D)** Cortical WM volume development for Chinese and U.S. male and female children. Note that Chinese children show higher cortical GM volume but lower WM volume compared to their U.S. peers, and the patterns of cortical GM and WM development for the two nationalities are in line with those of their global GM and WM development.

We tested for global brain and head volume changes over age, and differences in volume measurements between Chinese and U.S. children. We examined the effects of age, nationality, and gender on the development of head, intracranial, brain, GM, and WM MRI volumes. Figures 3–5 illustrate their development as a function of these factors. Table 3 lists detailed results of relevant statistical analyses and brief descriptions of key information indicated in Figures 3–5.

**Table 3:** **Hypothesis II: Head, intracranial, brain volumes, and GMand WM changes**.

	**Measurement**	**Nationality**	**Age**	**Nationality × Age**	**Gender**
Figures 3A,B	MRI head volume	*F*_(1)_ = 9.21^**^	*F*_(8)_ = 4.00^*^	*F*_(8)_ = 3.92^***^	*F*_(1)_ = 9.21^**^
		Overall US > CN but depends on age	Overall increase as age	US develops more dramatically than CN; CN >US before 11, US> CN after 11.	Overall Male >Female
Figures 3C,D	Intracranial volume	*F*_(1)_ = 12.26^***^		*F*_(8)_ = 3.73^***^	*F*_(1)_ = 9.05^**^
		Overall CN>US between 9 and 15		CN: inverted U peaking by 10 US: did not reveal a specific pattern.	Overall Male > Female
Figures 4A,B	Total brain volume	*F*_(1)_ = 29.49^***^		*F*_(8)_ = 2.52^*^	*F*_(1)_ = 8.88^**^
		CN > US who finally caught up by 16		Inverted U for CN and US, but peaking earlier for CN.	Male > Female, especially for US
Figures 4C,D	Total GM volume	*F*_(1)_ = 6.67^**^	*F*_(8)_ = 9.24^***^	*F*_(8)_ = 2.98^**^	
		Overall CN > US	Overall decrease for CN and US	Inverted U for CN and US, but peaking earlier for US. US > CN before 10, CN > US after 10	
Figures 4E,F	Total WM volume		*F*_(8)_ = 5.36^***^	*F*_(8)_ = 3.97^***^	*F*_(1)_ = 5.18^*^
			Overall increase for both CN and US	CN>US before 10, US>CN after 10	Male > Female for CN after 10
Figures 5A,B	Cortex GM	*F*_(1)_ = 42.79^***^	*F*_(8)_ = 7.57^***^	*F*_(8)_ = 2.06^*^	
		CN > US	Overall decrease for both CN and US	Inverted U for CN and US, but peaking earlier for US.	
Figures 5C,D	Cortex WM	*F*_(1)_ = 25.06^***^	*F*_(8)_ = 6.20^***^	*F*_(8)_ = 6.10^***^	
		US > CN after 13	Overall increase for both CN and US	US > CN after 13, and develops more dramatically	

Significant level:

* p < 0.05;

** p < 0.01;

*** p < 0.001;

empty cell refers to non-significant results.

Figure 3A–D shows the global head and intracranial volume changes for Chinese and U.S. children. Results showed that (1) the development of MRI head volume showed different patterns between the Chinese and U.S. children, which leads to a significant interaction between nationality and age; (2) differences were also found in the intracranial volume changes between Chinese and U.S. children, and Chinese children's intracranial volume was shown to be larger than U.S. cohorts; (3) the head and intracranial volumes were larger for males than for females, especially among U.S. children.

Figures 4A–E shows the development of total cerebral, global GM and WM volumes for Chinese and U.S. participants as a function of age, nationality, and gender. First, developmental patterns were different for total brain, GM, and WM volumes. Overall, there was a significant decrease in total GM volume and an increase in total WM volume for both the Chinese and U.S. children. Global GM volume peaked earlier for the U.S. than for the Chinese children (Figure 4C). There were also significant gender differences. The U.S., but not Chinese, male children showed larger intracranial volume than females; whereas Chinese, but not U.S., male children showed larger GM volume than females.

Figures 5A–D shows the development of GM and WM in the cortex for Chinese and U.S. children. The development patterns of cortical GM and WM for Chinese and U.S. children mostly agreed with their global GM and WM development. For example, cortical GM development showed an overall decline and an inverted U pattern for both Chinese and U.S. children peaking at different ages. The Chinese children had greater GM volume in the cortex than U.S. cohorts (Figures 5A,B); cortical WM development showed an overall increase, and older U.S. children and adolescents revealed larger cortex WM volume and a more dramatic increase than age-related Chinese cohorts (Figures 5C,D). These differences in developmental patterns were reflected in a significant interaction of age and nationality for cortical GM and WM volume (Table 3). No main effect of gender was found for cortical GM and WM development (Figures 5B,D).

### Hypothesis III: regional GM intensity and volume changes

**Figure 6:**
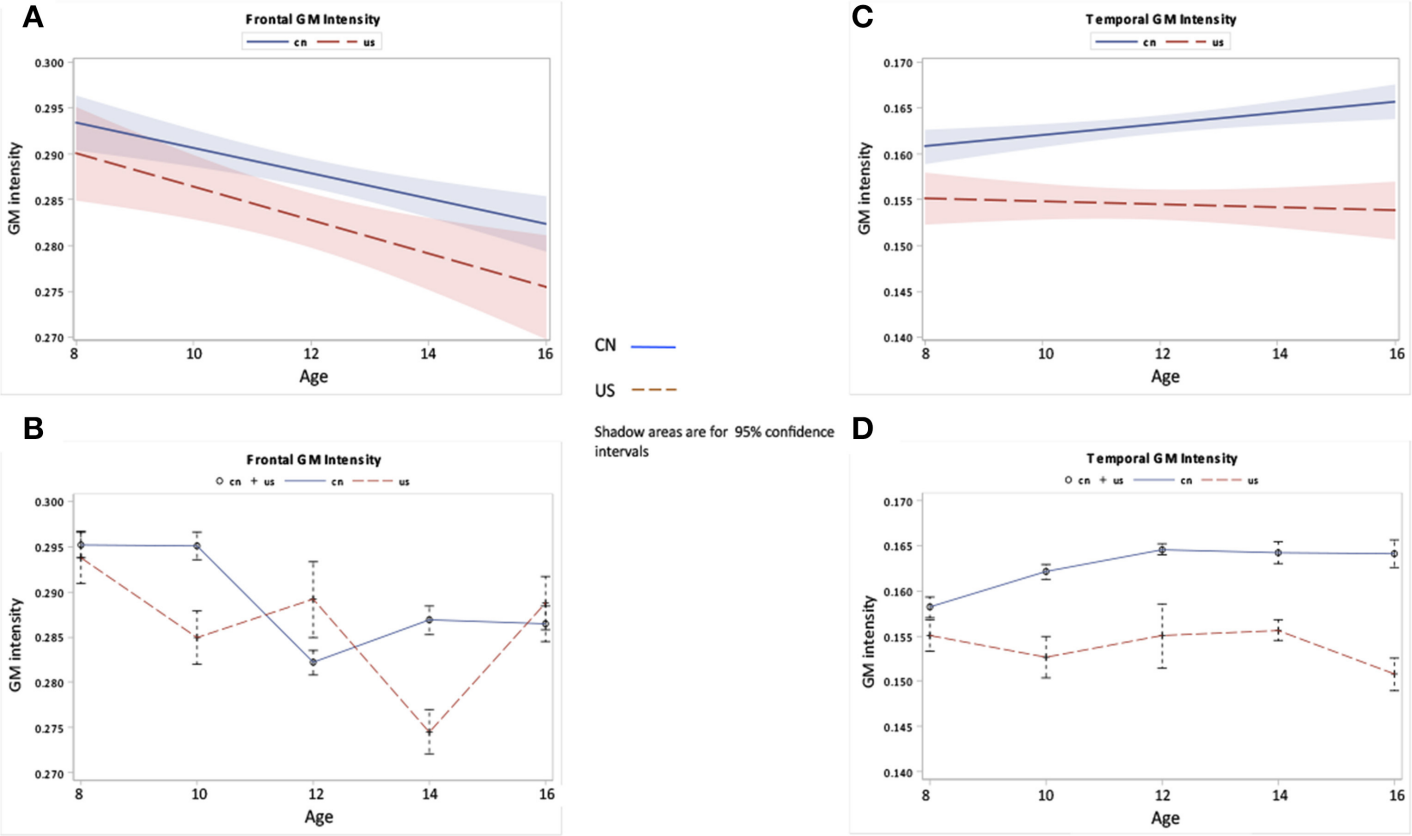
**Frontal and temporal GM changes as a function of age and nationality**. **(A,B)** Frontal GM development over ages for Chinese and U.S. children. **(C,D)** Temporal GM development over ages for Chinese and U.S. children. The top panel **(A,C)** shows the regression lines with 95% confidence intervals (CIs) for the development of GM, while the bottom panel **(B,D)** shows the GM development patterns with average and standard errors for each age group.

**Figure 7:**
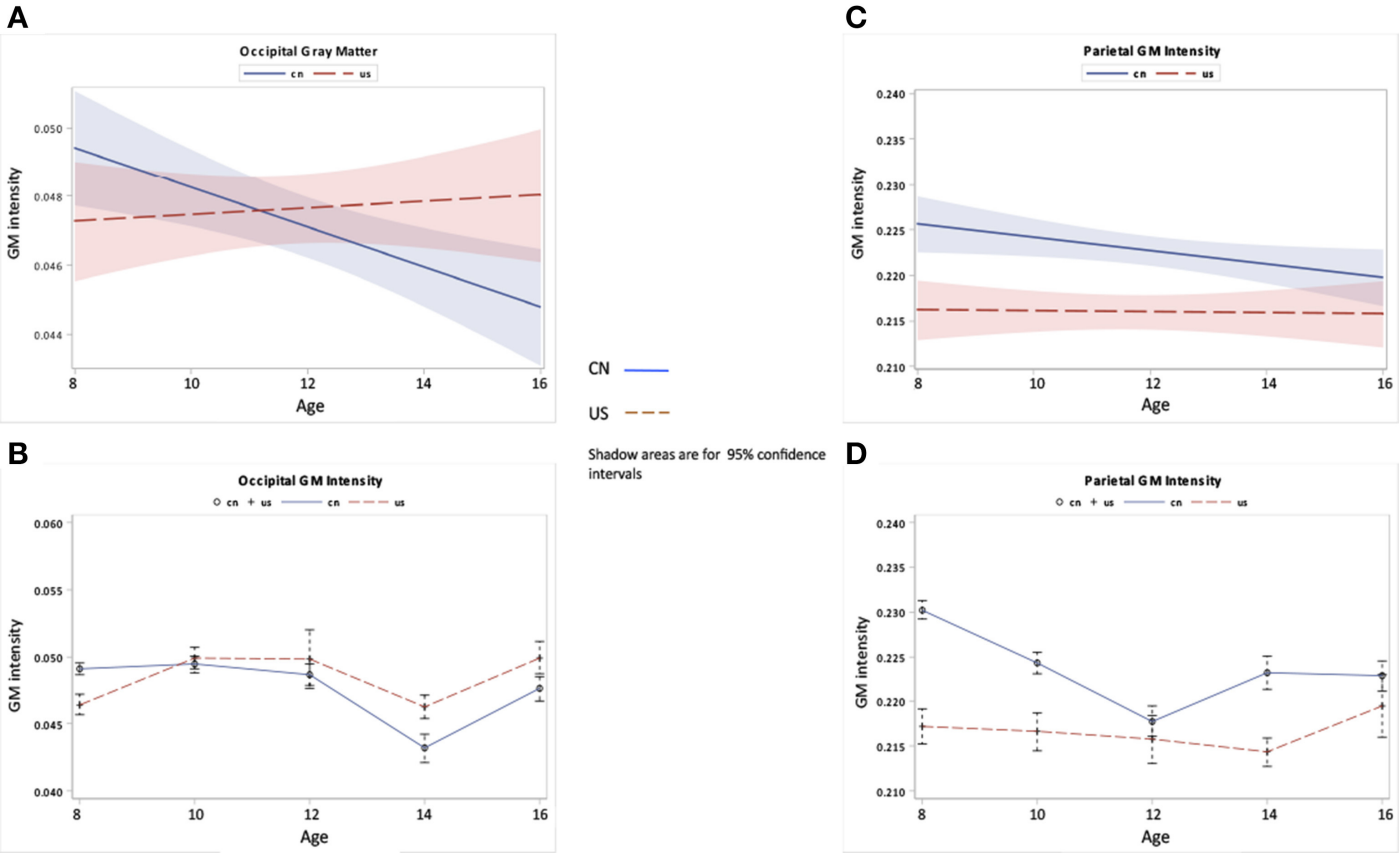
**Occipital and parietal GM changes as a function of age and nationality**. **(A,B)** Occipital GM development over ages for Chinese and U.S. children. **(C,D)** Parietal GM development over ages for Chinese and U.S. children.

**Figure 8:**
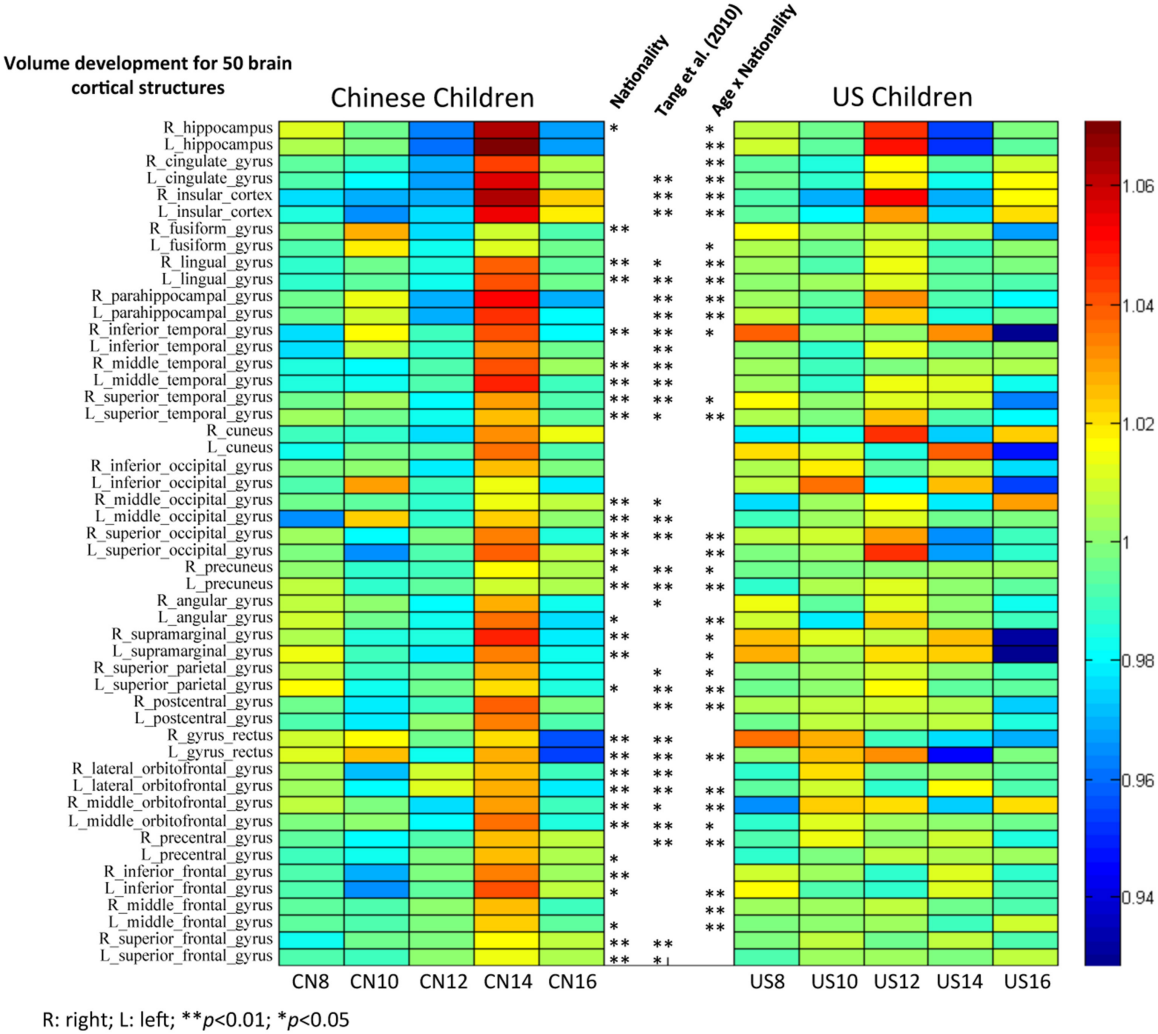
**The volume comparisons of 50 brain cortical structures between Chinese and U.S. children brains**. Subjects were grouped with two years increment. Mean volume proportion for the 50 brain structures were calculated for Chinese and U.S. children separately. Each single cell/grid stands for the ratio of the volume for a particular brain structure for that age group to the average for all age groups. Stars stand for the significant nationality effect found for particular structures. Stars on the left indicate the results for comparisons between Chinese and U.S. children from our study, and stars on the right indicate the results from Tang et al. (2010) with adults.

**Figure 9:**
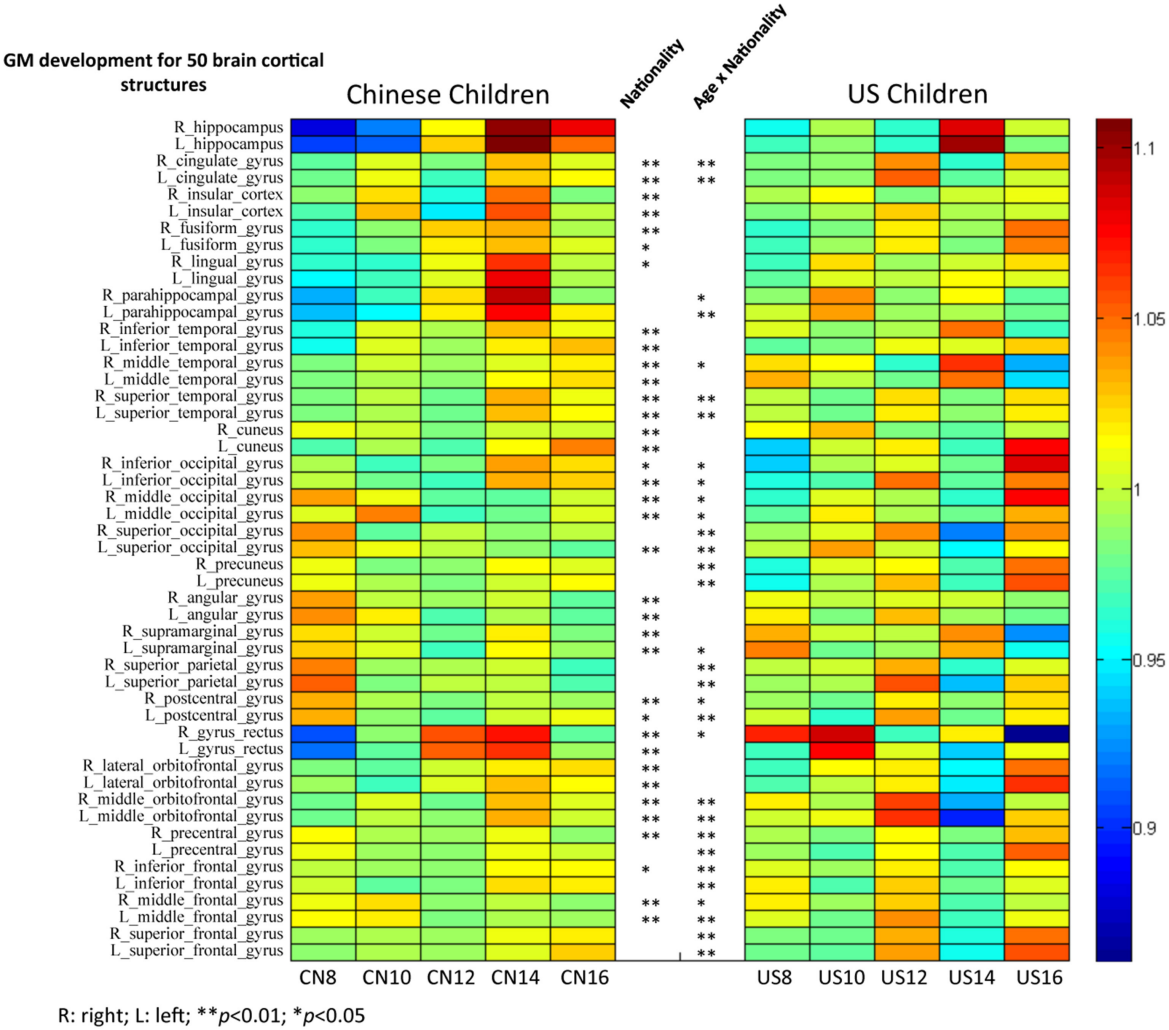
**The GM intensity comparisons of 50 brain cortical structures between Chinese and U.S. children brains**. Analyses used to make this figure were similar to those making Figure 8.

The third hypothesis tested GM changes as a function of age and nationality for four primary cerebral lobes including the frontal lobe, temporal lobe, occipital lobe, and parietal lobe (Figures 6, 7), total volume changes for 50 cortical brain segments (Figure 8), and the GM volume for 50 cortical brain areas (Figure 9). Statistical analyses results for GM development for four primary cerebral lobes are listed in Table 4.

**Table 4:** **Hypothesis III: Regional GM intensity and volume changes**.

	**Measurement**	**Nationality**	**Age**	**Nationality × Age**
Figures 6A,B	Frontal GM		*F*_(4)_ = 9.39^***^	
			Overall decrease as age	
Figures 6C,D	Temporal GM	*F*_(1)_ = 68.72^***^		*F*_(4)_ = 4.07^*^
		CN > US		CN increased from childhood to 12 followed by a plateau; US increased and peaked at around 14
Figures 7A,B	Occipital GM		*F*_(4)_ = 3.34^*^	
			Show decrease from 10 to 14 for CN and US, but no clear patterns.	
Figures 7C,D	Pariatal GM	*F*_(1)_ = 24.88^***^		
		CN > US		

Significant level:

* p < 0.05;

^**^p < 0.01;

*** p < 0.001;

empty cell refers to non-significant results.

Figures 6, 7 show the regional GM changes for the mean volume changes (Figures 6B,D, 7B,D) and regression lines for regional GM development (Figures 6A,C, 7A,C). GM in the frontal lobe showed an overall decline for both Chinese and U.S. children (Figures 6A,B), which was seen in a significant age effect. Temporal-lobe GM showed different developmental patterns for Chinese and U.S. children. Chinese children's temporal lobe GM increased from childhood to about 12 years followed by a plateau; whereas, it increased from childhood to 14 years for U.S. children (Figures 6C,D). The volume of the GM in the temporal lobe was larger for Chinese than for U.S. children. GM in the occipital lobe changed over age for both Chinese and U.S. children (Figures 7A,B), but no significant difference were found between the two nationalities. Parietal-lobe GM showed a decrease over age for Chinese children, but not for U.S. children, and was significantly larger for Chinese children than U.S. children (Figures 7C,D).

Figure 8 shows the total volume development of 50 cortical structures separately for Chinese and U.S. brains. Mean volume proportions for the 50 regions were calculated separately for Chinese and U.S. children. Each cell represents the ratio of the volume of a particular brain structure for that age group to the average volume for all age groups. A ratio greater than one (colors from green to red) indicates that the mean volume of that brain structure for that age group is greater than the average of all five age groups. In contrast, a ratio smaller than one (colors from green to blue) means that the mean volume of that brain structure for that age is less than the average. Thus, these color bars illustrated the development of all 50 regions separately for Chinese and U.S. children. The main effect of nationality and the interaction between age and nationality are illustrated in the middle of the figure between the two color bars. We included the adult results from Tang et al. (2010) for comparison.

The majority of brain cortical structures increased from childhood to early adolescence for both Chinese and U.S. participants. However, it can be seen in Figure 8 that most cortical structures of the U.S. children developed more gradually than Chinese children, and they peaked earlier (around 12 years) than Chinese children's (around 14 years). Figure 8 also shows that Chinese and U.S. children's brains were significantly different in volume for many cortical structures (*p* < 0.01). Most of these regions were consistent with findings by Tang et al. (2010) comparing the brains of Chinese and U.S. adults.

Similar analyses were conducted on the development of GM volume in these 50 brain structures for Chinese and U.S. children (Figure 9). GM volume showed different patterns of development for Chinese and U.S. children, especially in occipital, parietal, and frontal regions. Chinese children showed clear regional differences such that temporal and occipital structures matured later than parietal and frontal structures. These trajectories were consistent with the regional GM developmental patterns shown in Figures 6, 7. Temporal structures showed similar developmental patterns for American and Chinese cohorts. A majority of these cortical structures showed a significant nationality effect (*p* < 0.01) between Chinese and U.S. children. No results from the adult literature were included because no comparable analyses were established for Chinese and U.S. adults.

### Methods question: scanner types at different sites

**Figure 10:**
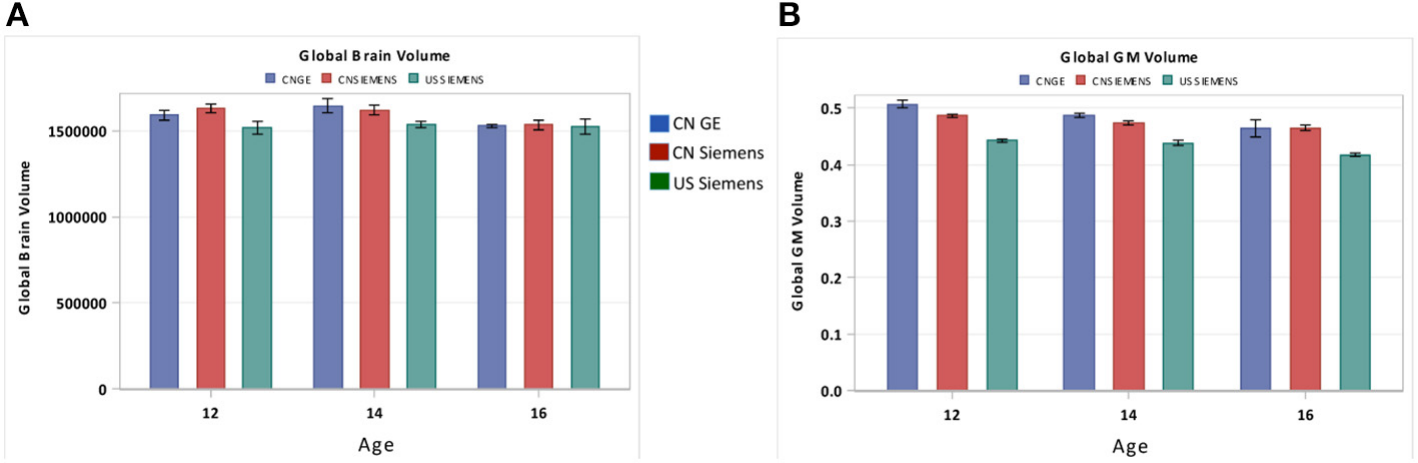
**Comparisons of the global (A) brain volume and the (B) GM volume between three different scanners including the GE scanner at China, the Siemens scanner at China, and the Siemens scanner at U.S. (USC-MCBI)**. Note that the Chinese scanners produced equivalent results. In addition, patterns of comparison between data from the US Siemens scanner and either of the two Chinese scanners were consistent with the results shown in Figures 4A,C.

A possible limitation of our study is the use of multiple scanners with different parameters. Although participants were scanned with similar field strength, potential biases resulted from utilizing different scanners might confound our findings. To address this issue, we compared the total brain volume and total GM volume for three different scanners including the Chinese GE scanner, the Chinese Siemens scanner, and the U.S. Siemens scanner at USC-MCBI. Figure 10 shows the comparison between these three scanners for both total brain volume (Figure 10A) and total GM volume (Figure 10B). From this comparison, we found that the Chinese scanners produced equivalent results. In addition, patterns of comparison between data from the US Siemens scanner and either of the two Chinese scanners were consistent with the results shown in Figures 4A,C. Thus, the differences found between Chinese and US participants are not expected to result from different scanners, but are due to real differences between the two nationalities.

## Discussion

This study compared head and structural brain development of Chinese children and adolescents with American age-related children and adolescents. The brains and heads of Chinese participants showed differences in morphological features (e.g., length, width, and height) compared with their American counterparts. Overall, Chinese children's brains and heads were shorter, wider, and taller than U.S. children's. The total head, intracranial, and cerebral volumes showed different patterns of change over age for the Chinese and U.S. children. Total head volume showed a linear increase with age for both Chinese and U.S. children, but U.S. children revealed a steeper slope than Chinese children. Intracranial and total brain volume showed an inverted U-shaped pattern for Chinese and U.S. children, but peaked at different ages. The overall volume of both GM and WM had similar developmental trajectories for Chinese and U.S. children, with some differences in the peak of the inverted-U function for the two nationalities. Regional GM comparisons showed differences in developmental patterns and volume between the two nationalities for the temporal and occipital lobes, while those for the frontal and parietal lobes were more similar between the nationalities. Finally, our detailed comparisons of 50 LPBA40 cortical structures between Chinese and U.S. children showed regional differences in both brain volume and developmental patterns.

Brain and head morphometric measurements confirmed our first hypothesis that Chinese and U.S. children and adolescents would be different in brain and head shape, size, and developmental patterns. These findings were consistent with results from comparisons of head and brain structures in Asian and North American adults (Lee et al., 2005; Tang et al., 2010). Specifically, Chinese children's brains were shorter, wider, and taller than age-related U.S. children, which mirror findings from direct comparisons of Chinese and North American adult brains (Tang et al., 2010). We did not find differences in the AC-PC distance between Chinese and U.S. children and adolescents, which we expected based on results reported by Tang et al. (2010). Perhaps the developmental patterns of the AC-PC distance in the two populations diverge after adolescence. The developmental trajectory of brain height was different between these two nationalities, while brain and head length and width showed similar developmental patterns. Future studies may investigate these features in younger children or even infants to better understand when these trajectories start to diverge. Differences in brain and head morphological features and developmental patterns between Chinese and U.S. children may be due to tissue level differences inside the brain.

Our results showed that global head volume increased linearly for both Chinese and U.S. children, but at different rates. The patterns of head volume development (Figure 3) were quite similar with those of head length development (Figure 2). The greater head volume found in the U.S. children might be due to their longer head length. Gender was also a factor: both Chinese and U.S. males showed greater head volumes than females. Chinese and American children showed different patterns of intracranial volume development; however, these patterns differed with those of head volume development. Chinese children showed larger intracranial volumes than U.S. cohorts, and males showed larger volumes than females. This dissociation may be caused by differences in the developmental trajectories of brain tissue (GM, WM, CSF) between these two populations. Since head volume includes other tissues (e.g., bones, skull), intracranial volume may be more informative in predicting brain development.

Our volumetric measurements of brain volume, GM, and WM development indicated that there are similarities and differences between Chinese and U.S. children's brain development. The global effects of age found in Chinese and U.S. participants were consistent with previous volumetric studies with Chinese and U.S. children (Giedd et al., 1996b, 1999; Guo et al., 2007; Lenroot et al., 2007). Specifically, our finding that the development of total cerebral volume followed an inverted U-shape for Chinese children peaking at early adolescence was consistent with previous findings with U.S. children (Lenroot et al., 2007). Chinese children's GM (inverted U) and WM (linear) developmental patterns (Figure 4) are similar to the findings for U.S. children reported in this study and previous research (Giedd et al., 1999; Lenroot et al., 2007). One difference regarding the patterns of the GM development is that GM development in the Chinese children peaked later than the U.S. children. No overall nationality effect was found on WM; however, there was an interaction of age and nationality on WM development, such that U.S. children had larger WM volume than Chinese children after 10 years of age. Our findings for cortical GM and WM development mostly mirror the patterns of overall GM and WM development, such that age and nationality affect the development of the cerebral cortex. The patterns of global GM and cortical GM development are more consistent with intracranial and brain volume development than WM development. One possible explanation is that due to the overall greater proportion of GM than WM proportion in the brain (see Figure 4), GM development has a stronger influence on children's brain developmental patterns. This may also explain why Chinese children had larger GM but smaller WM volumes than U.S. children and had larger intracranial volumes than U.S. cohorts.

The growth of brain lobes in Chinese children was partially comparable with that of North American children. Chinese temporal lobe GM development pattern mirrored previous findings from U.S. children, with linear increases from childhood (8 years) to adolescence (16 years). Parietal lobe GM development in Chinese children showed an overall reduction from childhood to adolescence, which is also consistent with previous findings from U.S. (Giedd et al., 1999) and Chinese children (Guo et al., 2007). Frontal GM development showed a linear decline in Chinese children, which is inconsistent with previous studies that reported nonlinear developmental patterns peaking around puberty in U.S. children (Giedd et al., 1999; Lenroot et al., 2007). Our U.S. participants failed to show clear patterns of temporal and parietal lobe GM development. A possible cause for this is the uneven number of male and female participants. The finding that Chinese children have higher levels of GM in temporal and occipital regions than American children may be the reason why Chinese children were found to have greater total brain volume and cortical GM volume than U.S. children.

Finally, we compared 50 cortical structures segmented using the LONI Probabilistic Brain Atlas (LPBA40, Shattuck et al., 2008). Volumetric comparisons between Chinese and U.S. children showed that more than half (30/50) of these brain structures were significantly different (*p* < 0.05) between Chinese and U.S. children's brains. The majority (21/30) of these distinct regions are consistent with Tang et al.'s (2010) study with adults. The gyri in the temporal, occipital, and orbitofrontal regions showed consistent differences in volume between Chinese and U.S. populations for both children and adults. Some structures (e.g., cingulate gyri, insular cortex) were not different for the Chinese and U.S. children in this study, but were different in adults (Tang et al., 2010). It is possible that some of these areas show differential growth in late adolescence or early adulthood, accounting for the differences between our data and Tang et al.'s (2010) results. The inverted U-shaped developmental patterns and different ages of peak volume for most of these brain structures are in accord with the differences seen in global GM and intracranial development between Chinese and U.S. children. Nationality had a significant effect on most of the GM volume comparisons for these 50 brain structures. These volumetric findings suggest that there is a need for population-specific (e.g., Chinese/Asian children) atlases in both structural and functional neuroimaging studies of brain structures.

We were unable to conduct an extensive examination of gender differences due to the unequal distribution of gender across age in the data of Chinese children. In the current study, we had limited numbers of female Chinese subjects for the first several age groups. Gender is an important factor in the delineation of brain structures for children and adolescents (Giedd et al., 1999; Sowell et al., 2004; Lenroot et al., 2007). Previous studies have shown that there are gender differences in brain development of U.S. children. Therefore, we would expect that future research with even number of males and females in each age might find interaction between gender, nationality, and age.

To the best of our knowledge, this study is the first that directly compares brain development between Asian and North American children. Our findings showed global and regional differences in both morphological and volumetric/anatomical brain development between the two populations. The Chinese children's brain was found to have different shape and size compared to U.S. children. Since we found many of these differences in our youngest age groups, this implies that these features already are different in younger children. Overall, Chinese children show similar global GM and WM development patterns to US children; however, Chinese children seem to have more GM but less WM (from puberty to adolescence) than US children. Measurements and comparisons for regional GM and 50 cortical structures support the detected global differences by showing detailed differences between these two populations. Both dissimilarities of genetics and environmental exposures might lead to these brain anatomical differences between Chinese and U.S. children; however, how much these factors contribute to the difference we found is unknown. Some of the brain areas that detected as being anatomically different in this study have shown robust functional differences in language processing between Chinese and Caucasian adult subjects (Kochunov et al., 2003; Kuo et al., 2003). Therefore, these anatomical differences detected between Chinese and U.S. children might lead to functional differences as well. Future research may investigate the effects of differences in brain anatomy on cognitive development (e.g., learning skills, language ability, attention, and memory development) in Chinese and American children and adolescents. Because Chinese children's brain structures mature at different rates than their American peers', they may have a different cognitive developmental trajectory, which would be an important consideration for East Asian educational systems. These anatomical differences between Chinese and U.S. children suggest the necessity for population-specific brain/head templates and atlas, and data processing and analyzing for neuroimaging research with Chinese/Asian children and adolescents.

### Conflict of interest statement

The authors declare that the research was conducted in the absence of any commercial or financial relationships that could be construed as a potential conflict of interest.
